# Enhanced Activity by NKCC1 and Slc26a6 Mediates Acidic pH and Cl^−^ Movement after Cardioplegia-Induced Arrest of db/db Diabetic Heart

**DOI:** 10.1155/2019/7583760

**Published:** 2019-09-08

**Authors:** Minjeong Ji, Seok In Lee, Sang Ah Lee, Kuk Hui Son, Jeong Hee Hong

**Affiliations:** ^1^Department of Physiology, College of Medicine, Gachon University, Lee Gil Ya Cancer and Diabetes Institute, 155 Getbeolro, Yeonsu-gu, Incheon 21999, Republic of Korea; ^2^Department of Thoracic and Cardiovascular Surgery, Gachon University Gil Medical Center, Gachon University, Incheon 21565, Republic of Korea; ^3^Department of Health Sciences and Technology, GAIHST, Gachon University, 155 Getbeolro, Yeonsu-gu, Incheon 21999, Republic of Korea

## Abstract

Diabetic heart dysfunctions during cardiac surgeries have revealed several clinical problems associated with ion imbalance. However, the mechanism of ion imbalance mediated by cardioplegia and a diabetic heart is largely unclear. We hypothesized that ion transporters might be regulated differently in the diabetic heart and that the differentially regulated ion transporters may involve in ion imbalance of the diabetic heart after cardioplegic arrest. In this study, we modified the Langendorff-free cardioplegia method and identified the involved ion transporters after cardioplegia-induced arrest between wild type and db/db heart. Enhanced expression of Na^+^-K^+^-2Cl^−^ cotransporter 1 (NKCC1) was observed in the db/db heart compared to the wild type heart. Enhanced NKCC1 activity was observed in the left ventricle of db/db mice compared to that of wild type after cardioplegia-induced arrest. The expression and activity of Slc26a6, a dominant Cl^−^/HCO_3_^−^ exchanger in cardiac tissues, were enhanced in left ventricle strips of db/db mice compared to that of wild type. The Cl^−^ transporting activity in left ventricle strips of db/db mice was dramatically increased as compared to that of wild type. Interestingly, expression of Slc26a6, as well as carbonic anhydrase IV as a supportive enzyme of Slc26a6, was increased in db/db cardiac strips compared to wild type cardiac strips. Thus, the enhanced Cl^−^ transporting activity and expression by NKCC1 and Slc26a6 in db/db cardiac tissues after cardioplegia-induced arrest provide greater insight into enhanced acidosis and Cl^−^ movement-mediated db/db heart dysfunction. Thus, we suggested that enhanced Cl^−^ influx and HCO_3_^−^ efflux through NKCC1 and Slc26a6 offer more acidic circumstances in the diabetic heart after cardioplegic arrest. These transporters should be considered as potential therapeutic targets to develop the next generation of cardioplegia solution for protection against ischemia-reperfusion injury in diabetic hearts.

## 1. Introduction

In cardiac surgeries under cardiopulmonary bypass and sporadic cardioplegia, damages may arise during intraoperative ischemia between multidose infusions of cardioplegia solutions or as a result of misdistribution of solutions distal to total coronary occlusions [1]. Additionally, potential reperfusion injuries occurred during each infusion of cardioplegia solutions and following removal of the aortic cross-clamp [1]. However, it is well known that ischemia-reperfusion (IR) injury during cardiac surgery is associated with increased mortality and morbidity [2]. Cardioplegia solutions provide protective effects regarding the myocardial global IR injury by maintaining the pH level during the ischemia [3, 4]. However, protective effect of cardioplegia on IR injuries was restrictive. Thus, combinational challenges of cardioplegic solutions and pharmacological agents to acquire further protective effect have been addressed [5–7].

Type 2 diabetes affects nearly four hundred million people worldwide. Between 2012 and 2030, there is a projected 69% and 20% increase in the number of adults with diabetes in developing and developed countries, respectively [1]. These patients show worse clinical outcomes following cardiac surgeries as compared with patients without diabetes [3, 8–10]. During ischemia, the accumulation of cytosolic H^+^ provides a greater driving force for the Na^+^-H^+^ exchanger (NHE). Accumulation of intracellular Na^+^ by NHE stimulates Ca^2+^ influx through the Na^+^/Ca^2+^ exchanger (NCX). Subsequent accumulation of cytosolic Ca^2+^ has been associated with the pathogenesis of cardiac dysfunction [1, 11, 12]. While cardioprotective methods have improved outcomes for the past several decades, there are still clinical problems associated with diabetic heart dysfunction, such as arrhythmias, apoptosis, and heart failure [13], which results in increased ion imbalance. It is known that IR injuries or cardioplegia-induced injuries following cardiac surgery are contributed by ion channel abnormalities in myocytes; there have been no studies that focused on whether ion channels work differently following cardioplegia-induced arrest in the myocardium of diabetic patients. The proper myocardial protection with specialized cardioplegia for diabetic patients is needed to decrease morbidity after cardiac surgeries.

The abundance and diversity of ion transporters of the heart are involved in the ion homeostasis. Therefore, studies that examine ion transporter changes in a diabetic myocardium after cardioplegia-induced arrest are essential. In this study, our goal was to address the following requests. Which transporters contribute to the pH modulation after cardioplegic arrest? And is there any difference between wild type (WT) and diabetic hearts? Although the ionic properties have been well established in cardiac myocytes [14, 15], whether the activities of ion transporters are maintained in cardioplegic arrested type 2 diabetic hearts remains unknown. Thus, we hypothesized that difference of the expression level and activity of ion transporters between WT and diabetic hearts will occur and that the difference may help to understand the mechanism of a diabetic heart after cardioplegic arrest. In the present study, we evaluated the differences in ion transporter function between db/db and WT mice following cardioplegia-induced arrest.

## 2. Material and Methods

### 2.1. Animals

An experimental mouse model of high glucose type 2 diabetes mellitus (db/db, BKS.Cg-Dock7m^+/+^Leprdb/J; male, 8 weeks of age) and normal glucose WT mice (C57BL/6, male, 8 weeks of age) was housed in the specific pathogen-free animal facility of the Lee Gil Ya Cancer and Diabetes Institute, Gachon University. The mice were housed in individually ventilated cages under controlled humidity (50%) and temperature (21.4°C). All experimental procedures for mouse maintenance and isolation of the heart from mice followed the Gachon University guidelines and were approved by the Gachon Animal Care and Use Committee of Gachon University (ACUC, LCDI-2016-0037).

### 2.2. Cardioplegia Infusion

All animal experiments were performed by a single cardiac surgeon. Animals were sedated with 5% isoflurane in an anesthetic chamber. We delivered cardioplegia by modifying the Langendorff-free cardioplegia method [16]. Following sedation, sternotomy and clam shell incision were quickly performed to expose the entire thorax; dissection around the aorta was carried out for aortic clamping. The right atrial auricle was excised to prevent left heart distension during cardioplegia infusion. A 23G needle was inserted in the left ventricle (LV); the needle insertion site was the apex of LV, and it was carefully chosen to avoid injuring coronary arteries. The above procedures were carried out prior to the start of heart fibrillation. The 23G needle was connected to either the cardioplegia or Regular solution. The histidine-tryptophan-ketoglutarate (HTK) solution at 5°C (Custodiol, Chemie GMBH, Alsbach-Hähnline, Germany) was administered using a roller pump at 1 mL/min per gram heart weight. During infusion, the ascending aorta was clamped with nontoothed DeBakey forceps. During cardioplegia infusion, care was taken to ensure that no LV distension was present and that coronary arteries were properly cleared by the cardioplegia solution. All procedures and infusion volume were identical between Regular and HTK solution infusions.

### 2.3. Cardiac Strip Preparation

**Figure 1:**
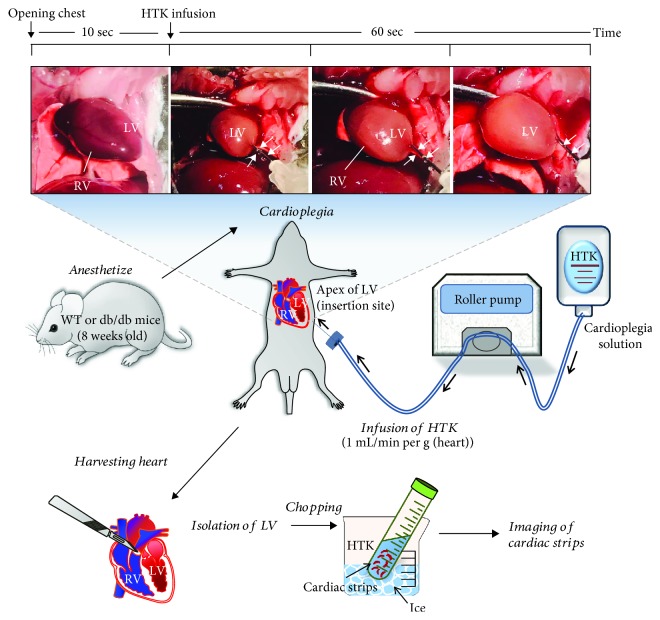
Langendorff-free cardioplegia method. Summary of the cardiac strip isolation followed by the Langendorff-free cardioplegia method. LV: left ventricle; RV: right ventricle. White arrows indicated the inserted needle for cardioplegia infusion.

Following cardioplegia infusion, the heart was harvested, and LV separation was performed. Briefly, minced cardiac strips (~100 × 150 *μ*m) were incubated in each HTK solution on ice. The separated LV was preserved in HTK or Regular solutions until needed for subsequent experiments. Imaging of isolated cardiac strips was completed within 1 hr. The compositions of HTK and Regular solutions are shown in Table 1. The Regular solution can be considered as HEPES-buffered physiological salt solution which consists of the same electrolyte composition like blood serum. To understand the cardioplegia delivery method and isolation of cardiac strip after harvesting the heart, we have schematically represented in Figure 1.

### 2.4. Analysis of Cl^−^-HCO_3_^−^ Exchanger (CBE) Activity of Cardiac Strips

Intracellular pH (pH_i_) was measured using the 2′,7′-bis-(carboxyethyl)-5-(and-6)-carboxyfluorescein (BCECF-AM, #0061, Teflabs, Austin, TX) at dual excitation wavelengths of 440 and 495 nm and an emission wavelength of 530 nm. Isolated cardiac strips on coverslips were incubated with 6 *μ*M BCECF-AM and 0.05% Pluronic F-127 (P3000MP, Invitrogen) in the chamber for 15 min at room temperature. The strips were perfused with cardioplegia solution for at least 5 min before measuring the pH_i_ at 37°C. The cardioplegic resting pH level was obtained from the initial BCECF fluorescence ratio (ratio = *F*_495_/_440_) of the first 60 sec of pH imaging during perfusion. The cardioplegic resting pH level was defined as the resting pH after cardioplegia-induced arrest. Slc26a6 is one of Cl^−^-HCO_3_^−^ exchangers (CBE) family. The CBE activity was measured in the presence of CO_2_-saturated HCO_3_^−^ media. Measurement was initiated by perfusing the tissues with the free Cl^−^-HCO_3_^−^ media (0 Cl^−^/HCO_3_^−^, Table 1). The determination of CBE activity was from the derivatives of the slopes (*Δ*pH_i_/sec) of the beginning of increase of pH_i_ in the free Cl^−^-HCO_3_^−^ media. The emitted wavelength was monitored with a CCD camera (Photometrics) attached to a microscope (Olympus, Japan); all images were analyzed with a MetaFluor system (Molecular Devices).

### 2.5. Analysis of Na^+^-K^+^-2Cl^−^ Cotransporter 1 (NKCC1) Activity in Cardiac Strips

NKCC1 activity was measured based on the rate of pH_i_ decrease, which was induced by intracellular NH_4_^+^ uptake [17]. Administration of 20 mM NH_4_Cl in the Regular solution mediated initial alkalization by NH_3_ diffusion; then, pH_i_ was decreased due to NH_4_^+^ pulse. In the second phase, the pH_i_ recovery rate following the NH_4_^+^ pulse was defined as the acidification rate (*Δ*pH_i_/sec). The initial linear acidification rate was fitted to a linear equation using the Origin software (version 8.0, OriginLab Inc.). The difference between acidification rates with or without bumetanide was used to calculate bumetanide-sensitive NKCC1 activity.

### 2.6. Analysis of Cl^−^-Transporting Activity by MQAE in Cardiac Strips

Intracellular Cl^−^ was measured using N-(ethoxycarbonylmethyl)-6-methaxyquinolinium bromide (MQAE, E3101, Thermo Fisher). The increased MQAE fluorescence represents decreased Cl^−^ concentration, while decreased fluorescence represents increased Cl^−^ concentration. LV strips on the coverslip were incubated with 5 mM MQAE for 30 min at room temperature and then washed with HTK or Regular solution until reaching the stabilization of the baseline signal. The MQAE fluorescence signal was recorded for at least 3 min to obtain the baseline signal, after which the perfusion solution was switched to free Cl^−^-HCO_3_^−^ media (0 Cl^−^/HCO_3_^−^, Table 1). This was followed by addition of HCO_3_^−^ solution (HCO_3_^−^, Table 1). The MQAE fluorescence was measured at 360 nm for excitation, and light emitted at 530 nm was collected with a CCD camera (Photometrics). The Cl^−^ transporting activity was determined from the derivatives of the slopes (MQAE fluorescence unit/sec) of the first 35-55 sec of fluorescence trace in free Cl^−^-HCO_3_^−^ media.

### 2.7. Confocal Imaging of Cardiac Tissues

For immunofluorescence studies, frozen cardiac tissue sections (10 *μ*m thick) were fixed with chilled methanol (for Slc26a6, ZO-1) or 4% paraformaldehyde (for NKCC1) for 10 min. Immunostaining was performed as previously described [18] using a 1 : 100 dilution of NKCC1 (ab59791, Abcam), ZO-1 (#33-9100, Thermo Fisher), and Slc26a6 (PA5-37970, Thermo Fisher) antibodies. Briefly, the bound antibodies were detected with Rhodamine (715-025-151, Jackson ImmunoResearch) and FITC (713-095-003, Jackson ImmunoResearch) (1 : 100 dilution). Coverslips were placed onto glass slides (Frost^plus^, Fisher) with DAPI-included Fluoromount-G™ (Electron Microscopy Sciences, Hatfield, PA), and images were analyzed using a LSM 700 confocal microscope through Fluo-view software (Carl Zeiss, Germany). The unstained sample was used as the negative control (NC).

### 2.8. Analysis of Na^+^/H^+^ Exchanger (NHE) Activity of Cardiac Strips

NHE activity was measured based on the recovery rate of pH_i_, induced by intracellular Na^+^ uptake in Regular solution as previously described [19, 20]. Briefly, after the decrease of the intracellular pH level by perfusing 20 mM NH_4_Cl solution, 0 Na^+^ solution was perfused. There was no recovery from acidification in 0 Na^+^ solution. And then, the intracellular pH_i_ was recovered by Na^+^-containing Regular solution, and the recovery rate was defined as NHE activity by measuring from the derivatives of the slopes of the first 35-55 sec of pH_i_ recovery trace.

### 2.9. Measurement of Serum Glucose Concentration

Mice were anesthetized with isoflurane (≈3% in air) by inhalation. The chest was opened to fully expose the heart, and blood was collected using 25G needle (Kovax-syringe 1 mL, Republic of Korea) from the heart. And then, collected blood was centrifuged for 15 min at 1,500 rpm. Then, the supernatant was collected, and the serum glucose level was measured using a blood glucose meter (Green Cross Mark, Republic of Korea).

### 2.10. Western Blotting

Cardiac tissues were isolated from mice and stimulated with the indicated cardioplegia solutions for 1 hr. Lysates of cardiac tissue homogenates were obtained with lysis buffer (containing (mM) 150 NaCl, 20 Tris, 2 EDTA, 1% Triton X-100, and a protease inhibitor mixture) and were treated as previously described [18]. 30 *μ*g denatured protein samples was subjected to SDS-PAGE. Proteins were visualized with NKCC1 (ab59191, Abcam), phosphoNKCC1 (#ABS1004, Millipore), Slc26a6 (ab172684, Abcam), CA IV (sc-74527, Santa Cruz Biotechnology), GAPDH (MA5-15738, Thermo Fisher), NHE1 (NBP1-76847, Novusbio), and *β*-actin (A3854, Sigma) antibodies using the enhanced luminescence (ECL) solution (Thermo Scientific). The intensity of protein band was normalized with that of *β*-actin as a protein loading control.

### 2.11. Isolation of Single Cardiac Myocytes for Di-8-ANEEPS Staining

Cardiac myocyte isolation was performed according to a previously generated protocol [16]. The LV from 8-week-old mice was injected with EDTA buffer containing (mM) 130 NaCl, 5 KCl, 0.5 Na_2_PO_4_, 10 HEPES, 10 Taurine, 10 glucose, 10 (2,3)-butanedione monoxime (BDM, Sigma Aldrich, B0753), and 5 EDTA. The heart was removed, and 10 mL EDTA buffer was injected into the left ventricle. Perfusion buffer (3 mL) was injected into LV containing (mM) 130 NaCl, 5 KCl, 0.5 Na_2_PO_4_, 10 HEPES, 10 Taurine, 10 glucose, 10 BDM, and 1 MgCl_2_. Collagenase buffer (30 mL) containing 0.5 mg/mL Collagenase type II (17101515, Thermo Scientific) and 0.05 mg/mL protease XIV (P5147, Sigma Aldrich) was injected into the LV until the heart is transparent and soft. Tissues were gently teased into 1 mm^3^ pieces and were transferred into 50 mL conical tubes. Stop buffer containing the perfusion buffer with 5% FBS was then added to cell suspension. Suspension was filtered with a 100 *μ*m strainer and was centrifuged for 3 min at 300×*g*. The supernatant was removed to isolate the cell pellet containing cardiac myocytes. To determine the T-tubule structure, 3 *μ*L of 10 mM di-8-ANEPPS (19541, Cayman, Ann Arbor, MI) and 2.5 *μ*L of 20% Pluronic F-127 were mixed. The freshly isolated cardiac myocytes were transferred to poly-L-lysine-coated cover slips and were incubated with mixed di-8-ANEPPS dye at 4°C (10 min). After staining, the mixed dye was washed with DMEM prior to confocal imaging.

### 2.12. Intracellular pH Calibration

Ratios of BCECF-AM (Teflabs) were converted to pH unit as described previously [21, 22]. Briefly, cardiac strips were incubated in the calibration solution (pH 5.5, 6.0, 6.5, 7.0, 7.5, 8.0, and 8.5) for 5 min at room temperature. The equation of the pH calibration curve was pH = pKa + log((*R*_max_‐*R*)/(*R*‐*R*_min_)) (*R*: ratio value of BCECF; *R*_max_: maximum ratio; *R*_min_: minimum ratio; pKa value of BCECF: 6.97). The BCECF fluorescence ratio was converted to the changes in the pH_i_ (*Δ*pH_i_) value, followed by the calibration curve.

### 2.13. DNA Transfection

Human SLC26A6 and human CA IV were developed in pCMV6-AC-mKate vectors; the original clones were provided by Dr. Shmuel Muallem (National Institutes of Health, Bethesda). Plasmid DNA transfection by Lipofectamine 2000 followed the manufacturer's protocol (11668019, Invitrogen) and was previously described [23].

### 2.14. Statistical Analysis

Results are expressed as the mean ± standard error of the mean (SEM). Significance was statistically determined by the analysis of variance in each experiment (^∗^*P* < 0.05, ^∗∗^*P* < 0.01, and ^∗∗∗^*P* < 0.001). Statistical differences between the mean values from the two sample groups were analyzed using Student's *t*-tests.

## 3. Results

### 3.1. Enhanced NKCC1 Protein in db/db Cardiac Tissues

**Figure 2:**
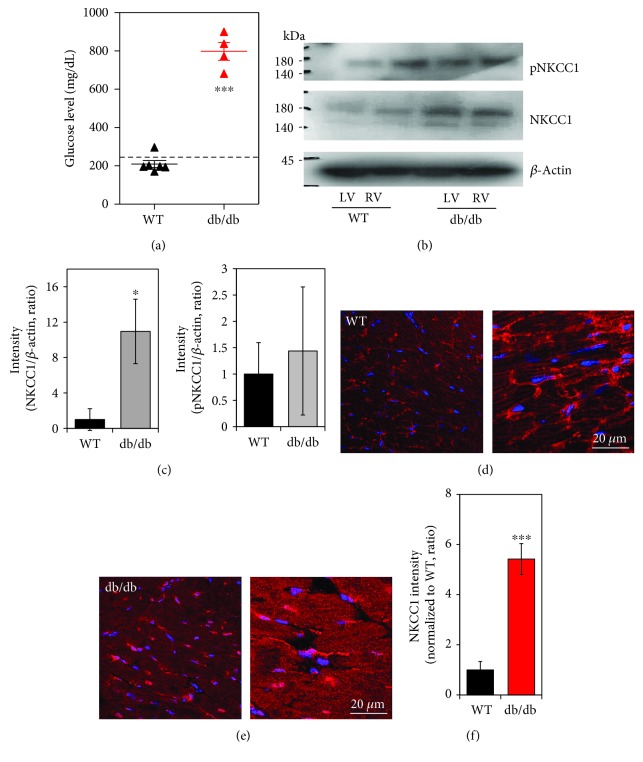
Enhanced NKCC1 protein in db/db cardiac tissues. (a) Serum glucose concentration in the WT (*n* = 6) and db/db (*n* = 4) mice (^∗∗∗^*P* < 0.001). Dotted line represents glucose 250 mg/dL. (b) Expression of phosphoNKCC1 (pNKCC1) and NKCC1 protein level in LV and RV cardiac strips of WT and db/db mice following cardioplegia-induced arrest. The *β*-actin was used as the loading control. (c) Analysis of band intensity of NKCC1 and pNKCC1 in LV. The bars show the mean ± SEM (*n* = 3, ^∗^*P* < 0.05). Immunolocalization of NKCC1 (red) and DAPI (blue) in LV of WT (d) and db/db mice (e). Right images of (d, e) are magnified. The scale bars represent 20 *μ*m. (f) Analysis of intensity of NKCC1 between WT and db/db LVs. The bars show the mean ± SEM (*n* = 5, ^∗∗∗^*P* < 0.001).

We first determined the diabetic mouse model with a high glucose level. The db/db mice provide a type 2 diabetes mouse model [24–26], and we confirmed the higher glucose level of db/db mouse serum (Figure 2(a)). We speculated that the [Na^+^]_i_ alteration by the NKCC1 cotransporter in the diabetic myocardium is higher than that in the normal myocardium, which may result in myocardial injury after cardioplegia-induced arrest. We measured differences in NKCC1 protein expression between wild type and db/db cardiac tissues. Enhanced NKCC1 protein expression was observed in LV^db/db^, whereas no difference in expression of phosphorylated NKCC1 (pNKCC1) protein was detected between wild type and db/db cardiac strips, and total NKCC1 protein was increased in db/db (Figures 2(b) and 2(c)). Next, we also examined expression of NKCC1 proteins in cardiac tissues with immunostaining. As shown in Figures 2(d)–2(f), NKCC1 expression was enhanced in db/db compared to wild type cardiac tissue.

### 3.2. Enhanced NKCC1 Activity in LV of db/db Cardiac Strips after HTK-Induced Arrest

**Figure 3:**
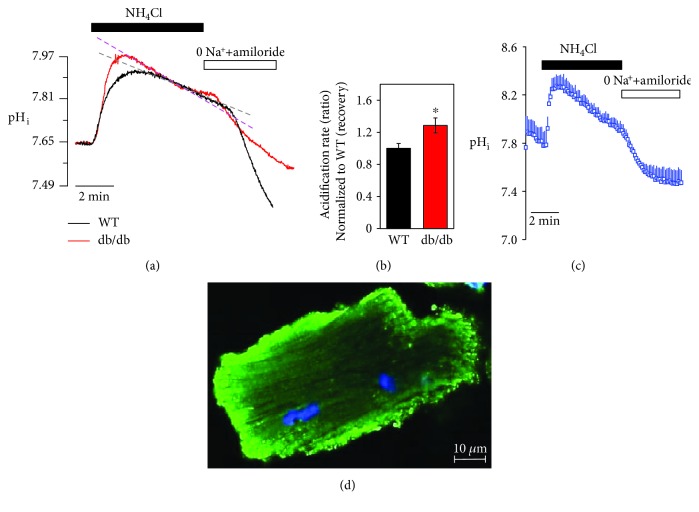
Enhanced NKCC1 activity in LV of db/db cardiac strips after HTK-induced arrest. (a) Changes in pH_i_ by NH_4_Cl pulse technique between WT and db/db cardiac strips. (b) Analysis of the acidification rate between HTK-arrested cardiac strips between WT and db/db (*n* = 5, ^∗^*P* < 0.05). (c) Changes in pH_i_ by NH_4_Cl pulse technique in isolated cardiac myocyte. (d) Image of ANEPPS staining (green) of isolated cardiac myocyte. The scale bar represents 10 *μ*m.

NKCC activity in cardiac tissue strips was examined with 20 mM NH_4_Cl pulse technique and determined via sensitivity to bumetanide, a selective NKCC1 inhibitor. Interestingly, cardioplegic arrest by HTK preserved the acidification property, not the initial alkalization, mediated by the NH_4_^+^ pulse (data not shown). Thus, we determined the recovery state of NH_4_^+^ pulse technique (Regular solution perfusion followed by HTK arrest for 1 min) to mimic the reperfusion followed by cardiac arrest. The alkalized NH_4_^+^ pulse was observed in LV^WT^ and LV^db/db^ (Figure 3(a)). The acidification rate of LV^db/db^ strips was increased compared to LV^WT^ strips (Figure 3(b)). To confirm the NH_4_^+^ pulse-induced changes in pH_i_, we measured pH_i_ in isolated single cardiomyocyte and compared pH changes of cardiac strips (Figure 3(c)). NH_4_^+^ pulse-induced pH trace between the cardiac strip and cardiomyocyte has the same pattern. Additionally, we determined the T-tubule structure in freshly isolated cardiac myocytes using the membrane-specific dye di-8-ANEPPS. Confocal images from cardiac myocytes stained with di-8-ANEPPS (green, Figure 3(d)) were obtained. The pH calibration curve of cardiac strips was applied to all changes in pH experiments (Supplementary [Supplementary-material supplementary-material-1]).

### 3.3. Increased Expression of Slc26a6 in db/db Cardiac Tissue

**Figure 4:**
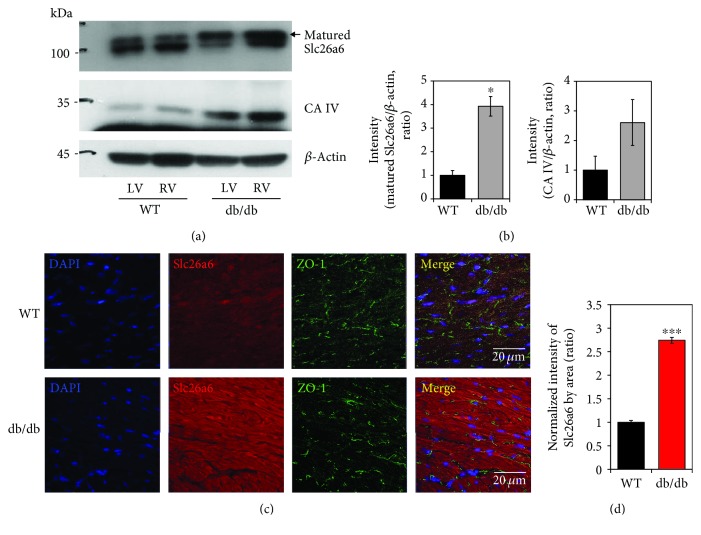
Increased expression of Slc26a6 in db/db cardiac tissue. (a) Protein expression of Slc26a6 and CA IV in cardiac tissues of WT and db/db mice. (b) Analysis of band intensity of mature Slc26a6 and CA IV in LV. *β*-Actin was used as the loading control. Bars present the mean ± SEM (*n* = 3, ^∗^*P* < 0.05). (c) Immunolocalization of Slc26a6 (red), intercalated disc marker ZO-1 (green), and nucleus (DAPI, blue) in cardiac tissues of WT and db/db mice. The scale bars represent 20 *μ*m. (d) Analysis of normalized intensity (total intensity/measuring area) of Slc26a6. Bars present the mean ± SEM (*n* = 4, ^∗∗∗^*P* < 0.001).

The Cl^−^/HCO_3_^−^ exchanger (CBE) mediates Cl^−^ uptake and acidification of pH_i_ through the HCO_3_^−^ efflux. Although AE3 is involved in CBE, we focused on Slc26a6, which dominantly expresses in the mouse heart compared to AE3 [27, 28]. To evaluate the expression of Slc26a6 in WT and db/db mice, we performed western blot analysis in WT and db/db cardiac tissues. Interestingly, Slc26a6 expression was enhanced in db/db mice (Figures 4(a) and 4(b)). In db/db cardiac tissues, the expression of CA IV was moderately increased, however, not statistically different (Figures 4(a) and 4(b)). Membrane-associated CA IV interacts with HCO_3_^−^ transporters to facilitate formation of HCO_3_^−^ transport metabolons [29]. We confirmed immunolocalization of Slc26a6 in cardiac tissues. The expression of Slc26a6 in LV^db/db^ was 2.7 times that of LV^WT^ (Figures 4(c) and 4(d)). These results suggested that the enhanced expression of the Slc26a6 facilitate Cl^−^ flux in db/db cardiac tissues.

### 3.4. Supportive Function of CA IV on SLC26A6 Activity and Enhanced CBE Activity in db/db Cardiac Strips

**Figure 5:**
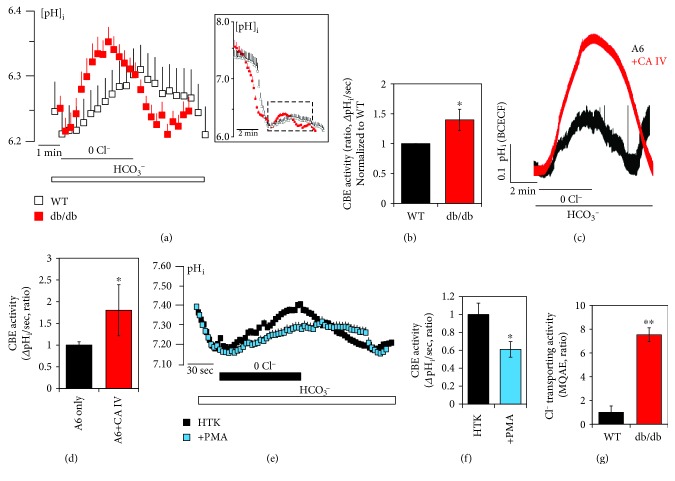
Supportive function of CAIV on SLC26A6 activity and enhanced CBE activity in db/db cardiac strips. (a) CBE activity was assessed by measuring changes in pH_i_ in LV cardiac strips of WT (open black square) and db/db (closed red square) mice following cardioplegia-induced arrest. Full image of CBE activity was represented in the square box, and magnified images were obtained from the dotted line. (b) Analysis of CBE activity. The slope of pH_i_ was measured as CBE activity in 0 Cl^−^. The bars show the mean ± SEM (*n* = 3, ^∗^*P* < 0.05). (c) Changes in pH_i_ by CBE activity of human SLC26A6- (A6-) overexpressed HEK293T cells with and without human CA IV. (d) Analysis of CBE activity. The slope of pH_i_ was measured in CBE activity in the 0 Cl^−^. The bars represent the mean ± SEM (*n* = 3, ^∗^*P* < 0.05). (e) Changes in pH_i_ by CBE activity with (closed blue square) and without (closed black square) 50 nM PMA and (f) analysis of CBE activity. The bars represent the mean ± SEM (*n* = 3, ^∗^*P* < 0.05). (g) Analysis of Cl^−^ transporting activity with MQAE technique between WT and db/db LV strips. The bars represent mean ± SEM (*n* = 4, ^∗∗^*P* < 0.01).

Whether CBE activity is maintained after cardioplegic arrest remains unknown. The CBE activity of LV^db/db^ strips was moderately increased as compared with that of LV^WT^ strips after the cardioplegic arrest (Figures 5(a) and 5(b)). Enhanced protein expression of Slc26a6 and CA IV will provide the increased CBE activity. Thus, we explored the mechanism underlying CA IV and SLC26A6-mediated changes *in vitro*. CA IV enhanced SLC26A6 activity (Figures 5(c) and 5(d)). Both CA IV and SLC26A6 did not interact with each other (data not shown); however, CA IV facilitated the Cl^−^/HCO_3_^−^ exchange activity of SLC26A6. To confirm the involvement of Slc26a6, protein kinase C agonist phorbol 12-myristate 13-acetate (PMA), known as a Slc26a6 inhibitor [30, 31], was added in cardioplegia solution. The CBE activity was inhibited by the treatment of PMA in cardiac strips (Figures 5(e) and 5(f)). To confirm the effect of Cl^−^ movement through NKCC and Slc26a6, changes in intracellular Cl^−^ concentration in LV^WT^ and LV^db/db^ strips were examined by the MQAE fluorescence quenching technique. MQAE fluorescence usually increases in the presence of 0 Cl^−^ media due to the decrease in intracellular Cl^−^ concentration [32]. Interestingly, the Cl^−^ transporting activity of LV^db/db^ strips was dramatically increased in 0 Cl^−^ media with MQAE fluorescence quenching technique (Figure 5(g)). These results suggested that the enhanced expression of Slc26a6 facilitates Cl^−^ transporting activity in db/db cardiac tissues after cardioplegia-induced arrest.

### 3.5. Activity and Protein Expression of NHE1 between WT and db/db after HTK-Induced Arrest

**Figure 6:**
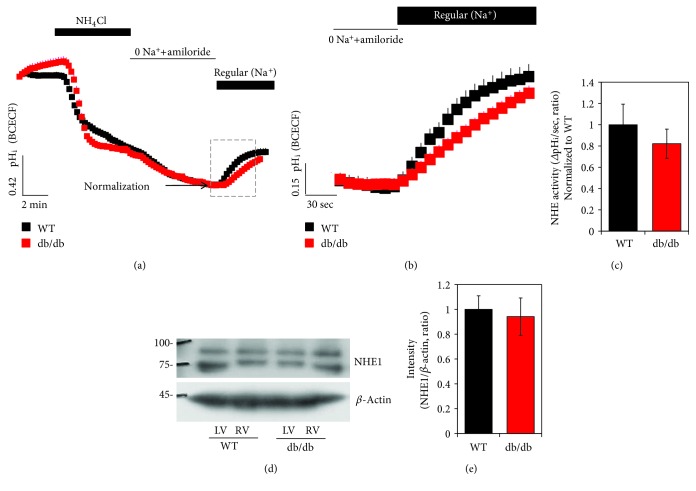
Activity and protein expression of NHE1 between WT and db/db after HTK-induced arrest. (a) NHE activity was assessed by NH_4_Cl pulse technique in LV cardiac strips of WT (closed black square) and db/db (closed red square) mice following cardioplegia-induced arrest. Full image of NHE activity was represented in the square box, and magnified images were obtained from the dotted line. (b) NHE activity in LV of WT after cardioplegia-induced arrest. (c) Analysis of NHE activity. The slope of pH_i_ was measured in NHE activity in the Na^+^-containing Regular solution. The bars represent the mean ± SEM (*n* = 3). (d) Expression of NHE1 protein in LV and RV cardiac strips of WT and db/db following cardioplegia-induced arrest. (e) Analysis of band intensity of NHE1 in LV. The *β*-actin was used as the loading control. The bars show the mean ± SEM (*n* = 3).

The Cl^−^/HCO_3_^−^ exchange facilitates Na^+^-loading, associated with Na^+^-dependent acid extrusion mechanisms such as NHE1, a major pH regulator [28]. We verified the role of NHE activity and protein expression after the cardioplegia-induced arrest. After the cardioplegia, there was no difference of NHE activity (Figures 6(a)–6(c)). The protein expression of NHE1 has no difference between LV^WT^ strips and LV^db/db^ strips (Figures 6(d) and 6(e)).

## 4. Discussion

We hypothesized that the diabetic myocardium is more vulnerable after cardioplegia infusion than the myocardium in the condition of normal glucose, as the various ion channels that are involved in [Na^+^]_i_ homeostasis are altered. Cation flux via NKCC following ischemia was shown to be expressively increased in type 1 diabetic hearts, and inhibition of NKCC revealed the reduced ischemic injury in diabetic hearts [33]. In this study, we found enhanced NKCC1 protein and increased NKCC1 activity in LV^db/db^. NKCC upregulation and its glycosylation disrupt Cl^−^ homeostasis [34]. Enhanced expression of NKCC1 in the db/db heart may provide synergistic dysregulation of Cl^−^ concentration in company with the enhanced Slc26a6. As enhanced CBE activity in LV^db/db^, the Cl^−^ transporting activity of Slc26a6 in LV^db/db^ was increased as compared with that of LV^WT^. In addition, activity of Slc26a6 in db/db may be further enhanced due to the involvement of CA IV. The kinetics of Cl^−^ flux was partly addressed in diabetic mice almost 40 years ago [35]. The Cl^−^/HCO_3_^−^ exchangers, encoded *Slc4a3* and *Slc26a6* genes, are expressed in heart tissue [27, 28, 36]. They are also called acid loaders and mediate HCO_3_^−^ efflux and Cl^−^ influx to induce acidification of pH_i_. Slc26a6 is known as a dominant transporter in the heart ventricle [27]. Although complicated regulatory mechanisms underlie the various ion transporters, we found enhanced expression of Slc26a6 and Cl^−^ transporting activity in the db/db heart, suggesting that effective Slc26a6 blockers may be efficient in modulating pH of type 2 diabetic hearts.

**Figure 7:**
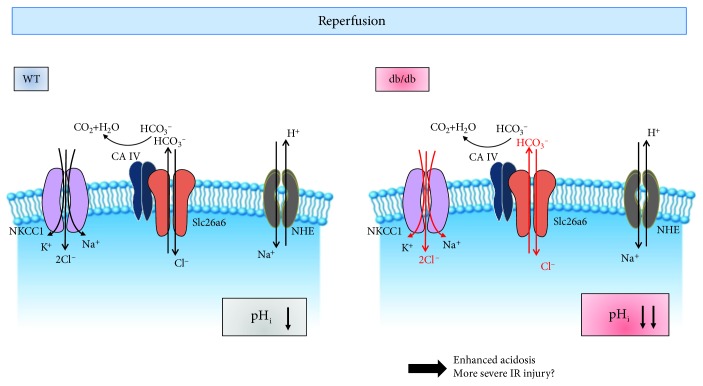
Schematic model of involvement of ion transporters followed by cardioplegia-induced arrest and ion imbalance in the db/db heart. Enhanced Slc26a6 and NKCC1 the in db/db heart may be involved in dysregulated pH_i_ and Cl^−^ modulation after cardioplegia-induced arrest. Abbreviation: NHE: Na^+^-H^+^ exchanger; Slc26a6: solute carrier transporter 26 family a6; NKCC1: Na^+^-K^+^-2Cl^−^ cotransporter 1; CA IV: carbonic anhydrase IV.

In this study, we addressed that the pH modulation after cardioplegic arrest was differentially regulated between WT and diabetic hearts. During the cardioplegia, the intracellular acidosis provides the primary source of ischemic reperfusion injury, and the mechanism is postulated in Figure 7. Based on our results, the db/db hearts were observed to increase NKCC, CBE, and Cl^−^ transporting activities caused by enhanced NKCC and Slc26a6 expression. It has been addressed that the enhanced expression and function of cardiac NKCC were observed during congestive heart failure and myocardial remodeling [37]. Enhanced NKCC activity of the db/db heart in the current study may also provide the pathological clue for the diabetic heart.

The HCO_3_^−^ modulation of Slc26a6 in the heart was revealed in several reports [27, 28]. It also has been addressed that the role of Slc26a6 was Cl^−^/oxalate exchanger to secrete oxalate rather than Cl^−^/HCO_3_^−^ exchanger in salivary glands [38]. Although we addressed enhanced Slc26a6 expression in the db/db heart, Cl^−^ window of Slc26a6 transporter needs to be clarified. It also has been carefully considered the enhanced Cl^−^ transporting activity in the db/db heart. Moreover, the physiological and pathological roles and cross-linked role of cardiac NKCC and Slc26a6 are currently unclear, and the mechanisms behind the Cl^−^ regulation cannot be concluded from the current results. Possibly, the elevated NKCC and Slc26a6 will provide the favorable circumstances to elevate Cl^−^ transporting activity in the db/db heart. In addition, the Cl^−^ influx and HCO_3_^−^ efflux by Slc26a6 may facilitate intracellular acidification during cardioplegia-induced arrest in the diabetic myocardium.

The results of the current study revealed several important limitations. For technical limitation, the current study was performed in normothermic temperatures, deviation from the cardiac surgery situation as Egar et al. mentioned [39]. However, the strength of our studies places in the fact that we modified the Langendorff-free method, which mimics immediately after aortic cross-clamp application and initial phase of cardioplegia-induced arrest during cardiac surgeries. In addition, the type 2 diabetes model (db/db mouse) with a high serum glucose level was used to evaluate the cardioplegia effect on the myocardium. Clinically, type 2 diabetes patients are more prone to cardiac diseases as compared to type I diabetes and require cardiac surgeries. Since the pathophysiological meaning of type 1 and 2 diabetes and their effect on the myocardium are different, our study is relevant in a clinical setting. Our study results suggested that it is necessary to develop cardioplegia that is specialized for diabetic patients. As such, this study suggests that modulation of ion-transporting activity in the db/db heart may be an effective strategy for preventing cardiac damage including acidosis and edema in diabetic patients after cardioplegia infusion.

## Figures and Tables

**Table 1:** Composition of solutions (mM).

	Regular	0 Ca^2+^	HTK	HCO_3_^−^	0 Cl^−^/HCO_3_^−^	0 Na^+^
NaCl	140	140	15	120	—	—
KCl	5	5	9	5	—	5
MgCl	1	1	4	1	—	1
CaCl_2_	1	—	0.015	1	—	1
HEPES	20	20	—	2.5	2.5	2.5
D-Glucose	10	10	—	10	10	10
Histidine	—	—	198	—	—	—
Tryptophan	—	—	2	—	—	—
Ketoglutarate	—	—	1	—	—	—
Mannitol	38	38	30	38.4	38.4	38.4
NaHCO_3_	—	—	—	25	25	—
Na-gluconate	—	—	—	—	120	—
Ca-gluconate	—	—	—	—	0.5	—
K-gluconate	—	—	—	—	5	—
MgSO_4_	—	—	—	—	1	—
NMDG-Cl	—	—	—	—	—	125

## Data Availability

All data and figures used to support the findings of this study are included within the article.

## Supplementary Materials

Supplementary Materials Supplementary Figure 1: pH calibration curve for LV cardiac strips at pH 5.5, 6.0, 6.5, 7.0, 7.5, 8.0, and 8.5. Ratios of BCECF-AM (Teflabs) were converted to pH unit as described previously [21, 22]. Briefly, cardiac strips were incubated in the calibration solution (pH 5.5, 6.0, 6.5, 7.0, 7.5, 8.0, and 8.5) for 5 min at room temperature. The equation of the pH calibration curve was pH = pKa + log((*R*_max_‐*R*)/(*R*‐*R*_min_)) (*R*: ratio value of BCECF; *R*_max_: maximum ratio; *R*_min_: minimum ratio; pKa value of BCECF: 6.97). The BCECF fluorescence ratio was converted to the changes in pH_i_ (*Δ*pH_i_) value, followed by the calibration curve.

## References

[1] Muraki S., Morris C. D., Budde J. M., Zhao Z. Q., Guyton R. A., Vinten-Johansen J. (2003). Blood cardioplegia supplementation with the sodium-hydrogen ion exchange inhibitor cariporide to attenuate infarct size and coronary artery endothelial dysfunction after severe regional ischemia in a canine model. *The Journal of Thoracic and Cardiovascular Surgery*.

[2] Opie L. H. (1991). Postischemic stunning--the case for calcium as the ultimate culprit. *Cardiovascular Drugs and Therapy*.

[3] Chambers D. J., Fallouh H. B. (2010). Cardioplegia and cardiac surgery: pharmacological arrest and cardioprotection during global ischemia and reperfusion. *Pharmacology & Therapeutics*.

[4] Dobson G. P., Faggian G., Onorati F., Vinten-Johansen J. (2013). Hyperkalemic cardioplegia for adult and pediatric surgery: end of an era?. *Frontiers in Physiology*.

[5] Camara A., An J., Chen Q. (2003). Na+/H+ exchange inhibition with cardioplegia reduces cytosolic [Ca2+] and myocardial damage after cold ischemia. *Journal of Cardiovascular Pharmacology*.

[6] Nardi P., Vacirca S. R., Russo M. (2018). Cold crystalloid versus warm blood cardioplegia in patients undergoing aortic valve replacement. *Journal of Thoracic Disease*.

[7] Yeh C. H., Chen T. P., Wang Y. C., Lin Y. M., Fang S. W. (2010). AMP-activated protein kinase activation during cardioplegia-induced hypoxia/reoxygenation injury attenuates cardiomyocytic apoptosis via reduction of endoplasmic reticulum stress. *Mediators of Inflammation*.

[8] Feng J., Sellke F. (2016). Microvascular dysfunction in patients with diabetes after cardioplegic arrest and cardiopulmonary bypass. *Current Opinion in Cardiology*.

[9] Frolich O., Karmazyn M. (1997). The Na–H exchanger revisited: an update on Na–H exchange regulation and the role of the exchanger in hypertension and cardiac function in health and disease. *Cardiovascular Research*.

[10] Karmazyn M., Moffat M. P. (1993). Role of Na+/H+ exchange in cardiac physiology and pathophysiology: mediation of myocardial reperfusion injury by the pH paradox. *Cardiovascular Research*.

[11] Pike M. M., Luo C. S., Clark M. D. (1993). NMR measurements of Na+ and cellular energy in ischemic rat heart: role of Na(+)-H+ exchange. *American Journal of Physiology-Heart and Circulatory Physiology*.

[12] Xiao X. H., Allen D. G. (2000). Activity of the Na^+^/H^+^ exchanger is critical to reperfusion damage and preconditioning in the isolated rat heart. *Cardiovascular Research*.

[13] Daniels L., Bell J. R., Delbridge L. M. D., McDonald F. J., Lamberts R. R., Erickson J. R. (2015). The role of CaMKII in diabetic heart dysfunction. *Heart Failure Reviews*.

[14] Balse E., Boycott H. E. (2017). Ion channel trafficking: control of ion channel density as a target for arrhythmias?. *Frontiers in Physiology*.

[15] Eder P. (2017). Cardiac remodeling and disease: SOCE and TRPC signaling in cardiac pathology. *Advances in Experimental Medicine and Biology*.

[16] Ackers-Johnson M., Li P. Y., Holmes A. P., O’Brien S.-M., Pavlovic D., Foo R. S. (2016). A simplified, Langendorff-free method for concomitant isolation of viable cardiac myocytes and nonmyocytes from the adult mouse heart. *Circulation Research*.

[17] Evans R. L., Turner R. J. (1997). Upregulation of Na(+)-K(+)-2Cl- cotransporter activity in rat parotid acinar cells by muscarinic stimulation. *The Journal of Physiology*.

[18] Lee D. U., Shin D. M., Hong J. H. (2016). The regulatory role of rolipram on inflammatory mediators and cholinergic/adrenergic stimulation-induced signals in isolated primary mouse submandibular gland cells. *Mediators of Inflammation*.

[19] Hu L. F., Li Y., Neo K. L. (2011). Hydrogen sulfide regulates Na^+^/H^+^ exchanger activity via stimulation of phosphoinositide 3-kinase/Akt and protein kinase G pathways. *The Journal of Pharmacology and Experimental Therapeutics*.

[20] Nishio S., Teshima Y., Takahashi N. (2012). Activation of CaMKII as a key regulator of reactive oxygen species production in diabetic rat heart. *Journal of Molecular and Cellular Cardiology*.

[21] Nehrke K. (2006). Intracellular pH measurements in vivo using green fluorescent protein variants. *Methods in Molecular Biology*.

[22] Rochon P., Jourdain M.´., Mangalaboyi J. (2007). Evaluation of BCECF fluorescence ratio imaging to properly measure gastric intramucosal pH variations in vivo. *Journal of Biomedical Optics*.

[23] Hong J. H., Muhammad E., Zheng C. (2015). Essential role of carbonic anhydrase XII in secretory gland fluid and HCO3 (-) secretion revealed by disease causing human mutation. *The Journal of Physiology*.

[24] Cavaghan M. K., Ehrmann D. A., Polonsky K. S. (2000). Interactions between insulin resistance and insulin secretion in the development of glucose intolerance. *The Journal of Clinical Investigation*.

[25] Gallo L. A., Ward M. S., Fotheringham A. K. (2016). Once daily administration of the SGLT2 inhibitor, empagliflozin, attenuates markers of renal fibrosis without improving albuminuria in diabetic *db/db* mice. *Scientific Reports*.

[26] Leibel R. L., Chung W. K., Chua S. C. (1997). The molecular genetics of rodent single gene obesities. *The Journal of Biological Chemistry*.

[27] Alvarez B. V., Kieller D. M., Quon A. L., Markovich D., Casey J. R. (2004). Slc26a6: a cardiac chloride-hydroxyl exchanger and predominant chloride-bicarbonate exchanger of the mouse heart. *The Journal of Physiology*.

[28] Wang H. S., Chen Y., Vairamani K., Shull G. E. (2014). Critical role of bicarbonate and bicarbonate transporters in cardiac function. *World Journal of Biological Chemistry*.

[29] Schwartz G. J., Kittelberger A. M., Barnhart D. A., Vijayakumar S. (2000). Carbonic anhydrase IV is expressed in H(+)-secreting cells of rabbit kidney. *American Journal of Physiology-Renal Physiology*.

[30] Hassan H. A., Mentone S., Karniski L. P., Rajendran V. M., Aronson P. S. (2007). Regulation of anion exchanger Slc26a6 by protein kinase C. *American Journal of Physiology-Cell Physiology*.

[31] Lee D., Lee S. A., Shin D. M., Hong J. H. (2018). Chloride influx of anion exchanger 2 was modulated by calcium-dependent spinophilin in submandibular glands. *Frontiers in Physiology*.

[32] Jeong Y. S., Hong J. H. (2016). Governing effect of regulatory proteins for Cl^–^/HCO_3_^–^ exchanger 2 activity. *Channels*.

[33] Ramasamy R., Payne J. A., Whang J., Bergmann S. R., Schaefer S. (2001). Protection of ischemic myocardium in diabetics by inhibition of electroneutral Na+-K+-2Cl- cotransporter. *American Journal of Physiology-Heart and Circulatory Physiology*.

[34] Ye Z. Y., Li D. P., Byun H. S., Li L., Pan H. L. (2012). NKCC1 upregulation disrupts chloride homeostasis in the hypothalamus and increases neuronal activity-sympathetic drive in hypertension. *The Journal of Neuroscience*.

[35] Berglund O. (1981). Disturbed fluxes of Rb+(K+) and Cl- in islets of spontaneously diabetic mice (C57BL/KsJ-db/db). *Acta Biologica et Medica Germanica*.

[36] Kim H. J., Myers R., Sihn C. R., Rafizadeh S., Zhang X. D. (2013). Slc26a6 functions as an electrogenic Cl^–^/HCO_3_^–^ exchanger in cardiac myocytes. *Cardiovascular Research*.

[37] Andersen G. O., Øie E., Vinge L. E. (2006). Increased expression and function of the myocardial Na-K-2Cl cotransporter in failing rat hearts. *Basic Research in Cardiology*.

[38] Mukaibo T., Munemasa T., George A. T. (2018). The apical anion exchanger Slc26a6 promotes oxalate secretion by murine submandibular gland acinar cells. *The Journal of Biological Chemistry*.

[39] Egar J., Ali A., Howlett S. E., Friesen C. H., O’Blenes S. (2014). The Na^+^/Ca^2+^ exchange inhibitor SEA0400 limits intracellular Ca^2+^ accumulation and improves recovery of ventricular function when added to cardioplegia. *Journal of Cardiothoracic Surgery*.

