# Supplementary material for: Enhanced Activity by NKCC1 and Slc26a6 Mediates Acidic pH and Cl− Movement after Cardioplegia-Induced Arrest of db/db Diabetic Heart
Source: Mediators Inflamm. 2019 Sep 8;2019:7583760. doi: 10.1155/2019/7583760 (PMC6754936; doi:10.1155/2019/7583760)
Supplement: Supplementary Materials — Supplementary Figure 1: pH calibration curve for LV cardiac strips at pH 5.5, 6.0, 6.5, 7.0, 7.5, 8.0, and 8.5. Ratios of BCECF-AM (Teflabs) were converted to pH unit as described previously [21, 22]. Briefly, cardiac strips were incubated in the calibration solution (pH 5.5, 6.0, 6.5, 7.0, 7.5, 8.0, and 8.5) for 5 min at room temperature. The equation of the pH calibration curve was pH = pKa + log((Rmax‐R)/(R‐Rmin)) (R: ratio value of BCECF; Rmax: maximum ratio; Rmin: minimum ratio; pKa value of BCECF: 6.97). The BCECF fluorescence ratio was converted to the changes in pHi (ΔpHi) value, followed by the calibration curve. [file 7583760.f1.pdf]

Supplementary Figure

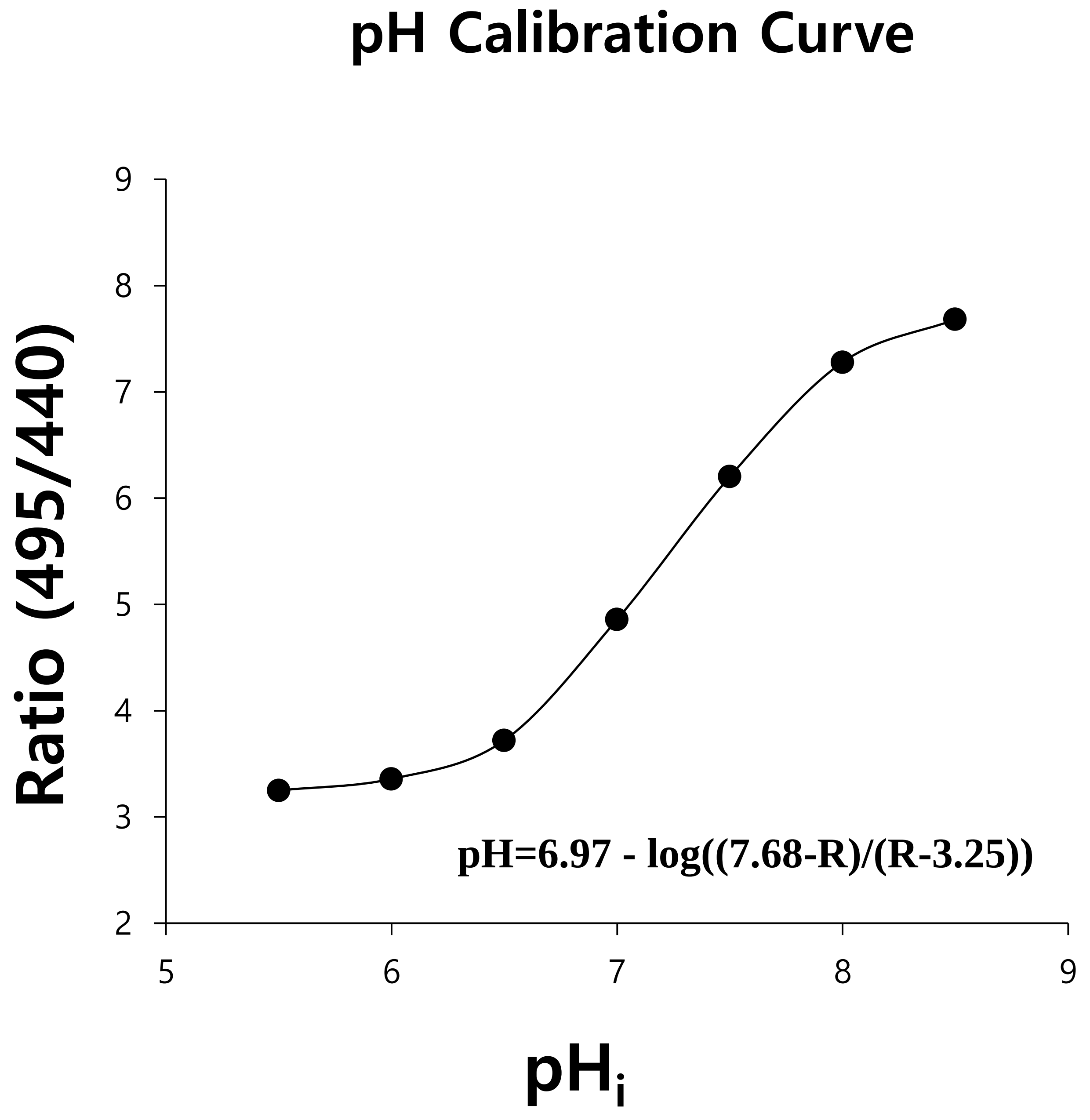

Supplementary Figure 1. pH calibration curve for LV cardiac strips at pH 5.5, 6.0, 6.5, 7.0, 7.5, 8.0, and 8.5. Ratios of BCECF-AM (Teflabs) were converted to pH unit as described previously (Nehrke, 2006; Rochon et al., 2007). Briefly, cardiac strips were incubated in the calibration solution (pH 5.5, 6.0, 6.5, 7.0, 7.5, 8.0, and 8.5) for 5 min at room temperature. The equation of pH calibration curve was pH = pKa + log((R_{max}-R)/(R- R_{min})) (R: ratio value of BCECF, R_{max}: maximum ratio, R_{min}: minimum ratio, pKa value of BCECF: 6.97). The BCECF fluorescence ratio was converted to the changes in pH_i (ΔpH_i) value, followed by the calibration curve.

* All supplementary data and figure used to support the findings of this study are included within the article
